# The influence of recovery period following a pre-load stimulus on physical performance measures in handball players

**DOI:** 10.1371/journal.pone.0249969

**Published:** 2022-03-31

**Authors:** Asmadi Ishak, Fui Yen Wong, Antoine Seurot, Scott Cocking, Samuel Andrew Pullinger

**Affiliations:** 1 Faculty of Sports Science & Coaching, Universiti Pendidikan Sultan Idris, Perak Darul Ridzuan, Malaysia; 2 Academy of Defence Fitness, National Defence University of Malaysia, Kuala Lumpur, Malaysia; 3 Département Physiothérapie, Swiss Olympic Medical Center, Meyrin, Suisse; 4 Sports Science Department, Aspire Academy, Doha, Qatar; 5 Sport Science Department, Inspire Institute of Sport, Vidyanagar, Dist. Bellary, India; University of Belgrade, SERBIA

## Abstract

The purpose of this research was to establish the optimal recovery duration following a pre-load stimulus on performance measures related to handball players. Seventeen senior male University handball players (mean ± SD: age 23.6 ± 2.3 yrs., height 1.79 ± 0.06 m and body mass 72.5 ± 10.7 kg) performed three experimental sessions. All sessions consisted of a standardised warm-up followed by a pre-load stimulus (HSR) back squats followed by a passive rest for either 4-min (PAP_4_), 8-min (PAP_8_), or 12-min (PAP_12_). Following the completion of the passive recovery, players then performed a countermovement jump (CMJ), a 20-m linear sprint and a modified agility t-test. The significance level was set at P < 0.05. There was a significant main effect of passive rest duration after the pre-load stimulus. The PAP_12_ condition improved CMJ scores (2.3–2.6%; effect size = small), 20-m linear sprint times (3.3–3.7%; effect size = small to moderate) and agility times (1.6–1.9%; effect size = trivial) compared to PAP_4_ and PAP_8_ conditions (P < 0.0005). Values of heart rate and rating of perceived exertion were also significantly lower during the PAP_12_ condition compared to the PAP_4_ and PAP_8_ conditions (P < 0.0005). A positive Pearson correlation was established between agility and CMJ for all conditions (P < 0.001). The findings provide novel data observing that a pre-load stimulus, followed by 12-min of recovery, results in greater maximal jump, sprint and agility measures when compared with a 4-min or 8-min recovery in male handball players.

## Introduction

Handball is an Olympic team sport, requiring players to intermittently perform maximal short duration actions. Strength and power are therefore important requisites to elite handball performance [1]. Time motion analysis in elite male handball athletes revealed players may cover a total distance of > 4km, performing 73 ± 32 high speed locomotive actions per game, including 14 ± 6 jumping actions and up to 31 ± 12 changes of direction per game, depending on playing position [2]. When focusing on physically preparing athletes to meet these in-game demands, warm-up routines involving specific components (Raise, Activate, Mobilise and Potentiate) are important in inducing specific physiological and biomechanical performance responses [3]. The “potentiate” stage of a warm-up is specifically associated with inducing delayed excitatory responses on myogenic and neurogenic systems [4]. This delayed excitatory response has been termed post activation potentiation (PAP) and is key in maximising power responses in team-sport athletes [5]. PAP typically involves performing a low number (3–6) of near maximal (> 85% 1RM) repetitions in muscle groups containing an abundance of fast twitch fibers, prior to competition. Myogenic adaptations to PAP may include the phosphorylation of myosin light chains, theoretically enhancing actin-myosin sensitivity to Ca^2+^ [4], whilst neurogenic responses may include enhanced synaptic efficacy, although this latter point remains contended [6].

Underpinning mechanisms relating to PAP-interventions have been explored in recent years [7] with methodologies eliciting a pre-load stimulus in team-sports well established [8–10]. Numerous variables have shown to affect the mechanisms influencing physical performance in team-sport athletes. One of the main variables affecting the efficacy of PAP is the rest-duration between the cessation of a pre-load stimulus, usually via performing a limited number of heavy strength repetitions (HSR) and the commencement of a performance task. A large amount of literature has investigated optimal rest-duration post-load stimulus on subsequent performance with recovery periods ranging from as little as 15-s up to 24-min [9, 11–14]. Findings suggest that team-sport have shown benefit to PAP with recovery durations ranging from 15-s [12], 4-min [15], 8-min [11], 12-min [5] or even beyond [16]. Further, it has been suggested that training status is a major determinant in pre-load stimulus realisation. Well-trained individuals may experience benefits from shorter recovery durations *versus* lesser-trained or recreationally trained individuals [16, 17], potentially affecting the efficacy of rest-durations across different populations. Other additional variables thought to affect the efficacy of PAP include muscle fiber type composition [18], conditioning contraction type [19] and sex [20].

Considering the large differences in methodologies used as pre-load stimulus protocol, varying recovery times and differences in population, comparing between studies is difficult. The pre-load stimulus protocol utilised, the performance variables assessed, and the training status of individuals affect findings. Little research has been conducted assessing pre-load stimuli on handball performance [8, 21, 22] and to the authors’ knowledge, one work to date has assessed the effect of rest duration on sport-specific PAP responses in handball athletes although this was not their main aim [8]. Therefore, the aim of this study was to investigate the optimal recovery duration following a pre-load stimulus (HSR) on performance measures related to handball players.

## Materials and methods

### Experimental approach to the problem

Each participant first completed two familiarisation sessions (detailed below), and thereafter, three experimental sessions consisting of a standardised warm-up followed by a pre-load stimulus (HSR) back squats and varying recovery times (4-min, 8-min or 12-min) after which they performed performance measures related to handball. These experimental sessions were counterbalanced in order of administration to minimise any potential learning effects [23], with a minimum of 72-h to ensure recovery between trials.

### Participants

Seventeen male University handball players (mean ± SD: age 23.6 ± 2.3 yrs., height 1.79 ± 0.06 m and body mass 72.5 ± 10.7 kg) volunteered to take part in this study. All players were recruited from the Universiti Pendidikan Sultan Idris handball team. Players were only selected if they met the inclusion criteria which were: at least 1 years of resistance training, had previous experience of squat exercises and habitually trained at any time of the day during a typical week. Players habitually trained three times per week and none had a history of recent musculoskeletal injuries before participating in this study. No one was taking any dietary supplements or pharmaceutical drugs that may affect performance during the study and all of them were free from illness during the study period. Verbal explanation of the experimental procedure was provided to each individual; this included the aims of the study, the possible risks associated with participation and the experimental procedures to be utilised. All players gave their written informed consent. The study was approved by the Human Ethics Committee of the Sport Science Department, Sultan Idris Education University, Malaysia and conformed to the Helsinki Declaration.

### Procedures

#### Protocol: Familiarisation session

Each participant performed a minimum of two familiarisation sessions under standard laboratory conditions (lighting and room temperature were 200–250 lux, 19–23°C), conducted over a 2-week period and finishing 1 week before the study commenced to minimize learning effects. During the familiarisation sessions 1 repetition maximum (1RM) for the back squat through a standardised 3-RM squat test following the guidelines set by the National Strength and Conditioning Association was determined for each participant [24]. Subjects were instructed to attempt three repetitions (with each repetition to 90° of knee flexion) of the chosen set load [25]. Upon completion of three successful repetitions, the weight was increased by ~ 15kg until the weight could no longer be lifted through the full range of motion (ROM). The 3-RM squat test required two to three attempts in order to be determined and only took place during the first familiarisation session. A five-minute recovery was provided between each set of three repetitions [26, 27]. An estimation of 1-RM was then determined using the table from [28] from the data collected during the 3-RM squat test. Following this, participants underwent familiarisation of the physical performance tests used in the study. These sessions ensured that participants were fully familiarised with the experimental conditions required for the study.

#### Protocol: Testing procedure

The subjects lived a “normal life” between sessions, slept at home at night and attended lectures and/or did light office work in the day. They were told to refrain from caffeinated beverages and from other training or heavy exertion for the 48 hours before the experiments or during them. On arrival compliance to the protocols’ sleeping, food intake and exercise restrictions were assessed verbally. Upon arrival, participants strapped on a heart rate monitor (Polar S710; Polar Electro Oy, Kempele, Finland) and undertook a 5-min general warm-up consisting of a self-paced jog followed by 2 sets of dynamic stretching of the lower musculature which compromised of 5 repetitions of bodyweight squats and 5 repetitions of lunge walks (each leg) over a 10-m distance. Once completed, subjects were asked to perform one set of 5 repetitions of back-squat at 85% 1-RM. This load has previously shown to successfully stimulate PAP [17, 29]. After undergoing the pre-load stimulus, subjects were instructed to passively rest for either 4-min (PAP_4_), 8-min (PAP_8_), or 12-min (PAP_12_). Following the completion of the passive recovery, participants were then asked to perform a countermovement jump, a 20-m linear sprint and a modified agility t-test. A rest of 2-min between trials and tests was included to minimise the effects of fatigue. Heart rate was measured throughout and ratings of perceived exertion [6–20] were measured throughout the test.

### Physical performance tests

#### Countermovement jump

The CMJ (a vertical jump test) was performed to assess the explosive power of the leg musculature. The test was performed on a force platform (Quattro jump: Kistler, Winterthur, Switzerland) and recorded with a sampling rate of 500Hz. The athletes were asked to perform the test with hands on hips and repeat this test 3 times, with a rest period of 30 s was provided in between each jump as previously described by [30]. They were instructed to execute the jumps using the correct technique, keeping their hands on their hips throughout the jump to minimize lateral and horizontal displacement and prevent any influence arm movements on jump performance [27]. Jumping height was measured as an estimate of the height change in the athlete’s center of mass, taking into consideration the total duration the athlete spends in the air with no ground contact. The highest CMJ recorded was used for further analysis. This test has previously been reported to be both valid and reliable [31].

#### Linear 20-m sprint test

Acceleration and maximum running speed were determined during the 20-m linear sprint test, a relevant performance parameter in team-based sports [32]. The athlete was required to run a single maximal sprint over a 20-m distance. Sprint times were recorded using timing gates (Microgate, Bolzano, Italy) with set 1-m apart, 1-m height and 1-m from the starting line. The position of the timing gate was standardized following the guidelines by the manufacturer. The starting position was standardized for each sprint, placing the dominant foot placed at the front. All athletes repeated the test 3 times with a 3 min recovery period to ensure results were reliable. The times of the best 20-m linear sprint was used for further analysis. This test has previously been reported to be both valid and reliable for use in team-sports [33, 34].

#### Modified agility t-test

**Fig 1:**
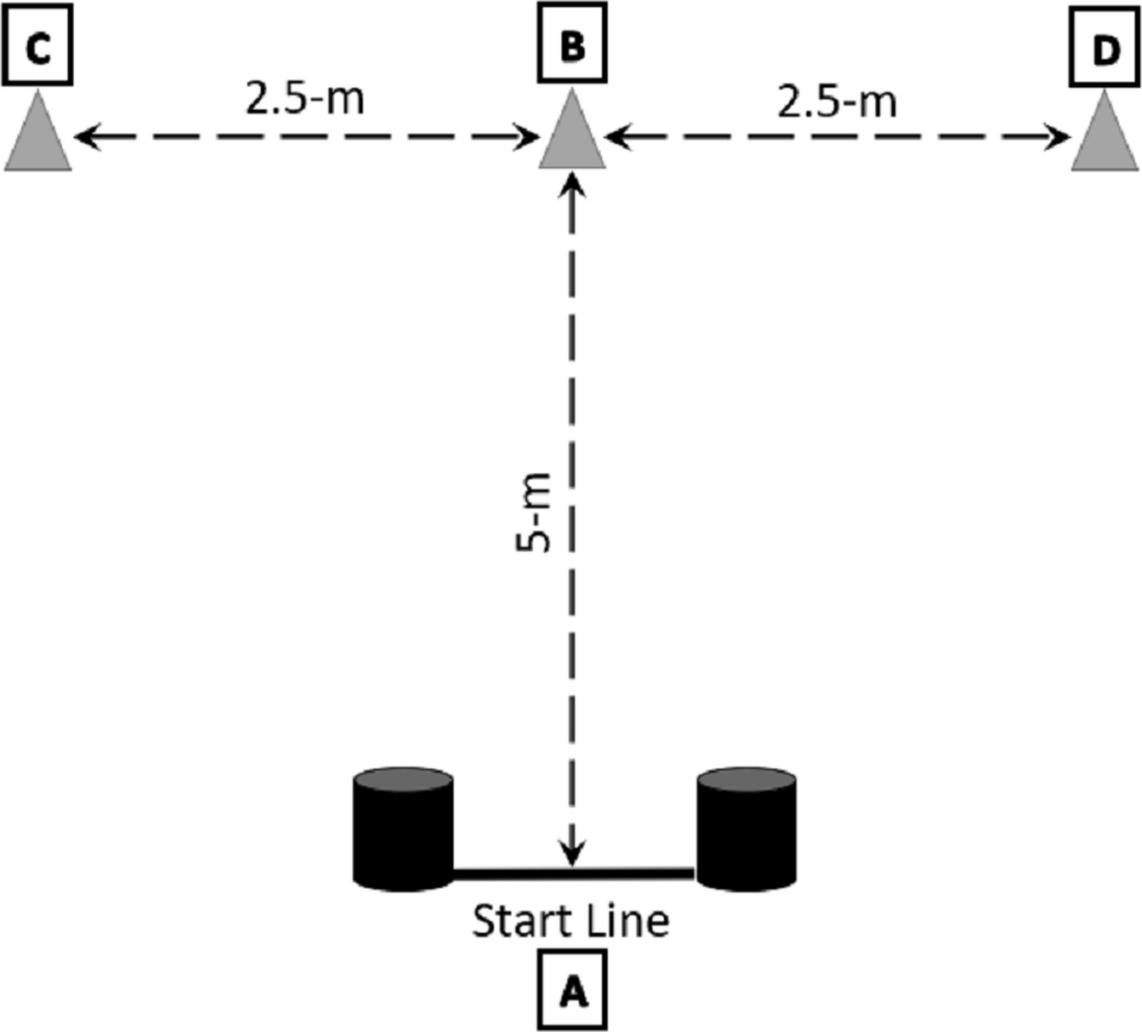
Illustration of the modified agility t-test assessment as used by Sassi et al. **[35].** The grey triangles indicate the cones around which the athletes must run and shuffle. The black cylinder indicates the placement of timing gates (Microgate, Bolzano, Italy), set 1-m apart, 1-m in height and 1-m from the pre-marked start line/finish line.

Agility facets involving acceleration, deceleration, and balance control were determined using a modified agility t-test as a predictor of handball performance (Fig 1). The t-test was administered using the modified protocol used by [35]. Four cones were used to mark the start/finish line (point A), the middle (point B) and the end-points (points C and D). The athlete was required to start with both feet behind the starting line (point A) and sprint forward 5-m and touch the cone (point B) with the right hand and then shuffle 2.5-m left to another cone (point C) and touch it with the left hand. Once the cone has been touched, the athlete must shuffle to the right 5-m to the furthest cone (point D), touch it with the left hand, and shuffle back 2.5-m to the middle cone (point B), touching the cone with the left hand, after which the athlete runs backwards for 5-m to the finish line (point A). Any time an individual failed to touch the base of the cone with his hand, crossed one foot in front of the other or did not face forward throughout the test, it was deemed unsuccessful and the test had to be repeated. Agility times were recorded using timing gates (Microgate, Bolzano, Italy) with set 1-m apart, 1-m height and 1-m from the starting line. The position of the timing gate was standardized following the guidelines by the manufacturer. The athlete performed 3 trials, with the best time used for subsequent analysis. This test has previously been reported to be reliable to assess agility [35].

### Statistical analysis

All data were analysed using Statistical Package for the Social Sciences (SPSS IBM, Chicago, IL, USA) version 26. Differences between conditions were evaluated using a one-way repeated-measures analysis of variance for all performance variables. A GLM with repeated measures were used for HR and RPE measures (condition [3 levels] x time [3 levels]). To correct violations of sphericity, the degrees of freedom were corrected in a normal way, using Huynh-Feldt (ε > 0.75) or Greenhouse-Geisser (ε < 0.75) values for ε, as appropriate. Graphical comparisons between means and Bonferroni pairwise comparisons were made where main effects were present. Effect sizes (ES) were calculated from the ratio of the mean difference to the pooled standard deviation. The magnitude of the ES was classified as trivial (≤ 0.2), small (> 0.2–0.6), moderate (> 0.6–1.2), large (> 1.2–2.0) and very large (> 2.0) based on guidelines from [36]. The results are presented as the mean ± the standard deviation throughout the text unless otherwise stated. Ninety-five percent confidence intervals are presented where appropriate and were corrected for between subject differences. The approach involves the conceptualisation of the trends over time for the ‘average person’ by normalising subject means and expressing all changes relative to the same mean. Following convention, the alpha level of significance was set at 5% where values P<0.05 have been referred to as ‘‘significant”. Values of “0.000” given by the statistics package are shown here as P < 0.0005 [37].

## Results

### Physical performance tests

**Fig 2:**
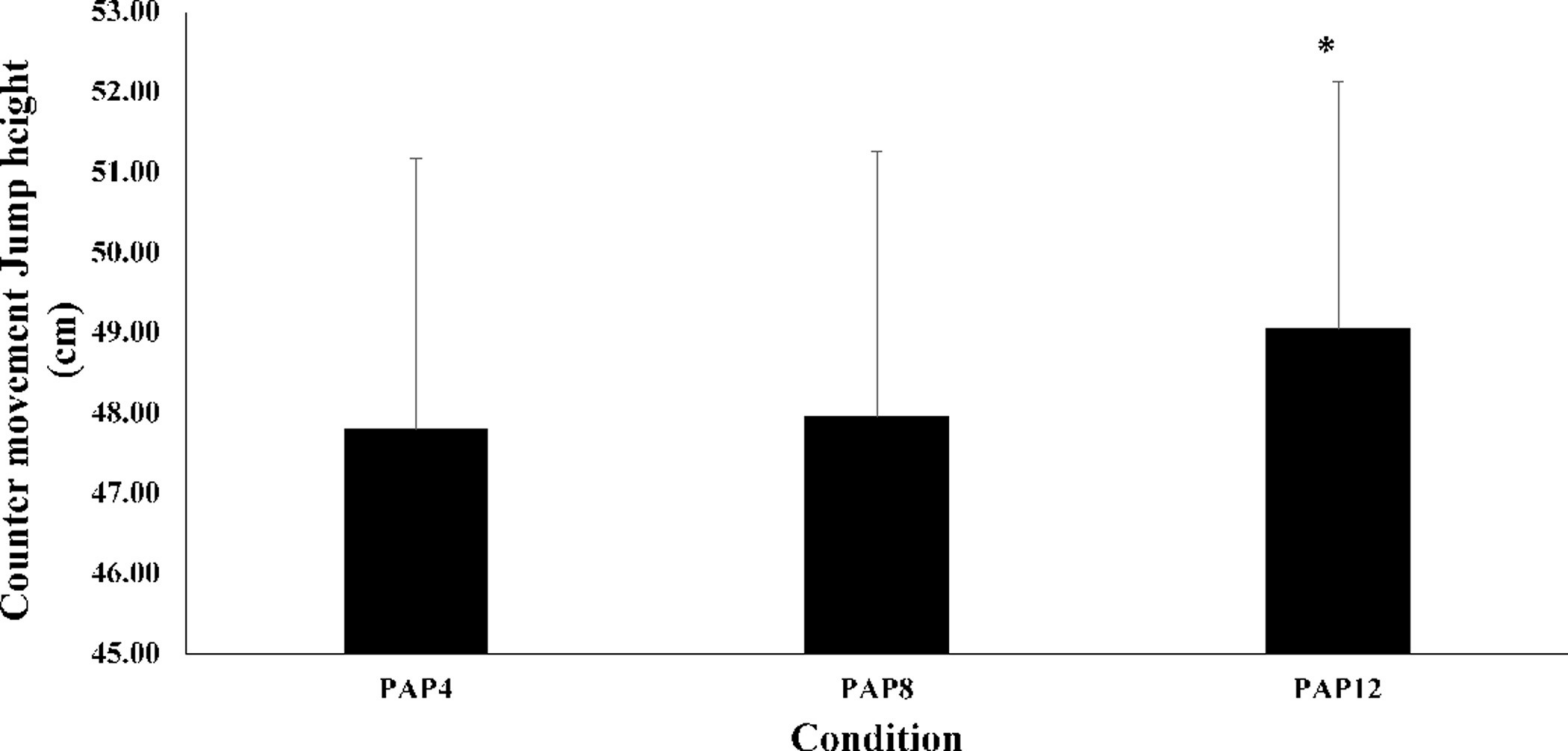
Mean ± SD for countermovement jump height (cm) for the PAP4, PAP8 and PAP12 conditions. *Denotes a statistical significance compared to other conditions (P < 0.0005).

There was a significant main effect of passive rest duration after the pre-load stimulus on CMJ scores (P < 0.0005; Table 1; Fig 2). Values for CMJ height in the PAP_12_ condition improved by 2.25% (mean difference = 1.08 (0.75, 1.41) cm, P < 0.0005, ES = 0.34, small) and 2.60% (mean difference = 1.25 (0.85, 1.65) cm, P < 0.0005, ES = 0.39, small) compared to the PAP_8_ and PAP_4_ conditions, respectively. No differences were observed in CMJ height between PAP_8_ and PAP_4_ (P = 0.433).

**Table 1:** Mean (± SD) values for physical performance variables and subjective measures in the three PAP conditions. **Statistical significance (*P* < 0.05) is indicated in bold.** The magnitude of the ES is classified as trivial (≤ 0.2), small (> 0.2–0.6) and moderate (> 0.6–1.2).

Variable	PAP_4_	PAP_8_	PAP_12_	Significance of main effects for condition	Significance of main effects for time	Interaction	ES (4–12)	ES (8–12)
**CMJ (cm)**	47.80 ± 3.37	47.97 ± 3.30	49.05 ± 3.09*	***P* < 0.0005**			0.39	0.34
**Agility (s)**	8.33 ± 0.87	8.31 ± 0.86	8.18 ± 0.87*	***P* < 0.0005**			0.18	0.15
**20-m sprint (s)**	3.52 ± 0.21	3.51 ± 0.20	3.40 ± 0.21*	***P* < 0.0005**			0.61	0.57
**RPE (6–20)**	14.2 ± 0.9	13.9 ± 0.8	13.2 ± 0.6	***P* < 0.0005**	***P* < 0.0005**	*P* = 0.988		
**HR (bpm)**	154 ± 4	153 ± 3	151 ± 3	***P* = 0.007**	***P* < 0.0005**	*P* = 0.970		

* Denotes a significant difference with PAP_4_ and PAP_8._

**Fig 3:**
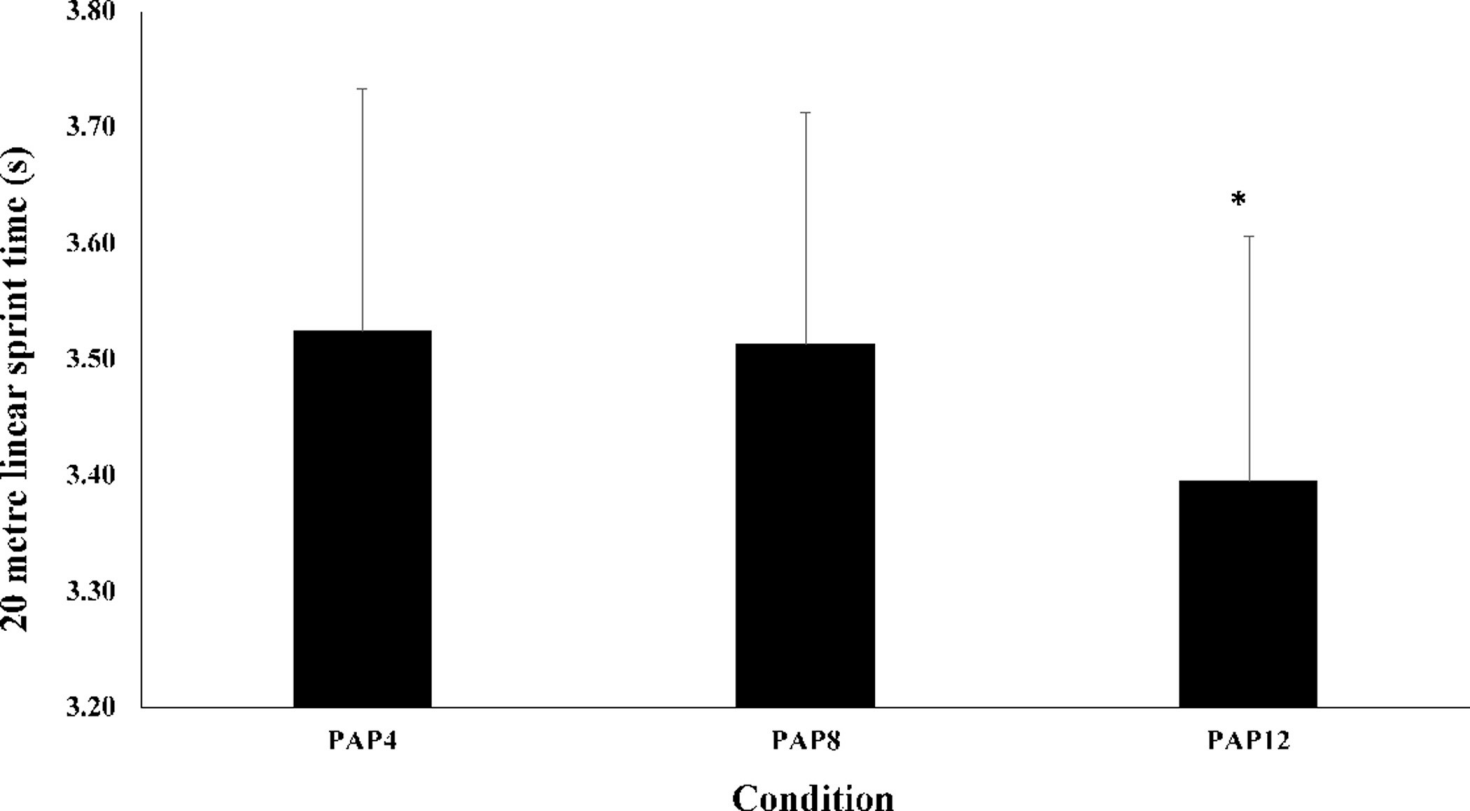
Mean ± SD for 20-m linear sprint times (s) for the PAP_4,_ PAP_8_ and PAP_12_ conditions. *Denotes a statistical significance compared to other conditions (*P* < 0.0005).

There was a significant main effect of passive rest duration after the pre-load stimulus on 20-m linear sprint times (P < 0.0005; Table 1, Fig 3). Values for sprint times in the PAP_12_ condition improved by 3.34% (mean difference = 0.12 (0.07, 0.16) s, P < 0.0005, ES = 0.57, small) and 3.65% (mean difference = 0.13 (0.08–0.17) s, P < 0.0005, ES = 0.61, moderate) compared to the PAP_8_ and PAP_4_ conditions, respectively. No differences were observed in 20-m linear sprint times between PAP_8_ and PAP_4_ (P = 0.999).

**Fig 4:**
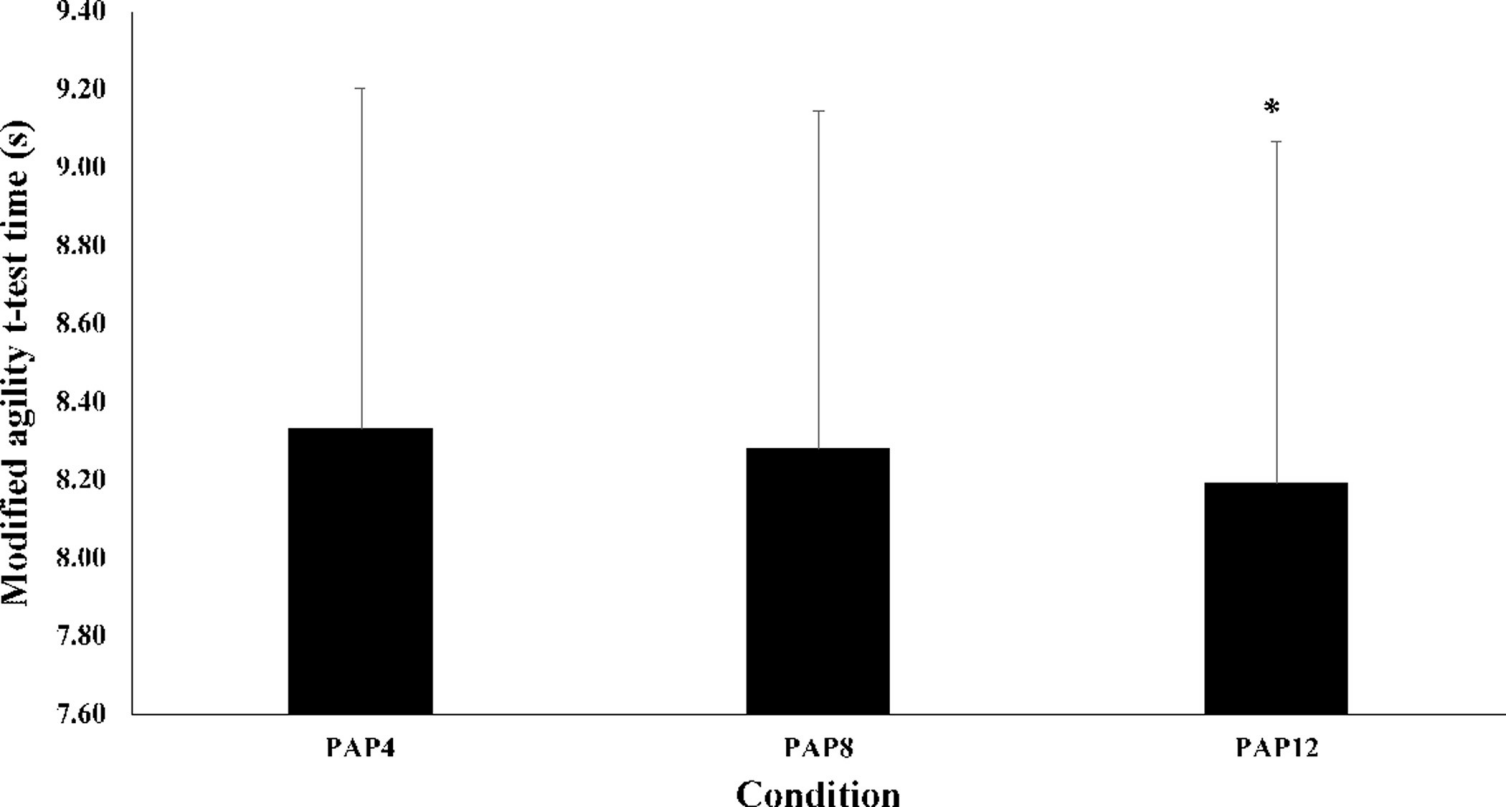
Mean ± SD for modified agility t-test times (s) for the PAP4, PAP8 and PAP12 conditions. *Denotes a statistical significance compared to other conditions (P < 0.0005).

There was a significant main effect of passive rest duration after the pre-load stimulus on agility times (P < 0.0005; Table 1, Fig 4). Values for agility times in the PAP_12_ condition improved by 1.55% (mean difference = 0.13 (0.10, 0.16) s, P < 0.0005, ES = 0.15, trivial) and 1.85% (mean difference = 0.15 (0.11, 0.20) s, P < 0.0005, ES = 0.18, trivial) compared to the PAP_8_ and PAP_4_ conditions, respectively. No differences were observed in agility times between PAP_8_ and PAP_4_ (P = 0.054).

A positive Pearson correlation between agility time and jump height was observed for the PAP_4_ condition (P < 0.001), PAP_8_ (P < 0.001) and PAP_12_ (P < 0.001). Sprint times displayed no correlation with agility time or jump height (P > 0.05).

#### Physiological parameters

HR values showed a main effect for condition and time (P < 0.0005). HR values were significantly lower in the PAP_12_ condition compared to the PAP_8_ (mean difference = 3 (2, 4) beats.min^-1^, P < 0.0005) and PAP_4_ (mean difference = 4 (3, 5) beats.min^-1^, P < 0.0005) conditions. HR responses were lower post-CMJ compared to post-agility (mean difference = 23 (-25, -21) beats.min^-1^, P < 0.0005) and post-sprint (mean difference = 53 (-54, -52) beats.min^-1^, P < 0.0005). HR responses were also significantly lower post agility compared to post-sprint (mean difference = 31 (-32, -29) beats.min^-1^, P < 0.0005). There was no significant interaction between time and condition (P = 0.988), with HR values increasing after each physical performance test irrespective of condition (Table 1).

RPE showed a main effect for condition and time (P < 0.0005). RPE values were significantly lower in the PAP_12_ condition compared to the PAP_8_ (mean difference = 1 (-1, 0), P < 0.0005) and PAP_4_ (mean difference = 1 (-1, 0), P < 0.0005) conditions. RPE values were lower post-CMJ compared to post-agility (mean difference = 2 (-3, -2), P < 0.0005^1^) and post-sprint (mean difference = 6 (-7, -6), P < 0.0005). RPE values were also significantly lower post agility compared to post-sprint (mean difference = 4 (-4, -3), P < 0.0005). There was no significant interaction between time and condition (P = 0.970), with RPE values increasing after each physical performance test irrespective of condition (Table 1).

## Discussion

The primary aim of this study was to investigate the optimal recovery duration following a pre-load stimulus on maximal physical performance measures, specific to the demands of handball. We demonstrate that university level handball athletes experience the greatest benefit in handball specific performances measures 12-min after a pre-load stimulus (heavy strength repetitions), *versus* either 4- or 8-min post pre-load stimulus. In addition to these performance benefits, both, rate of perceived exertion and heart rate were statistically lower when performance tests were performed 12-min after the initial pre-load stimulus.

### Discussion around main finding

Literature observations show the greatest recovery responses to a pre-load stimulus have ranged from as little as 15-s up to 12-min and beyond [9, 11–14] with no agreement as to the optimal amount of recovery time needed. This is likely due to recovery time between a pre-load stimulus and maximal performance capacity being highly dependent on numerous factors including the exercise stimulus, training status or even gender [11, 26]. The need for individual determination of the optimal recovery time from pre-load stimulus to competition, is therefore vital when trying to maximise readiness to perform. In humans, both fatigue and potentiation responses, although antagonistic in nature, can coexist [38], therefore whilst potentiation following a pre-load stimulus can elicit favourable enhancements in speed and power [39], fatigue can induce opposing responses [15]. In order to induce benefit from PAP, adequate recovery must take place [38].

Whilst our findings show the optimal recovery duration in university level handball athletes was 12-min post pre-load stimulus across all (jump, sprint, agility) performance tests, many elite team-sport athletes are relatively stronger in comparison to less-elite players [39]. Given greater muscle strength (1RM) is significantly correlated with shorter rest durations following a pre-load stimulus in order to elicit maximal PAP responses [17], an assessment in more elite players may hypothetically result in a shorter optimal duration such as PAP_4_, PAP_8_ or even < 4-min. Another consideration for analysis would be to investigate player position specific responses to PAP. For example, it is reported that goalkeepers are often larger and stronger than wingers [40] and secondly that backs and pivots may perform more high-intensity actions than wingers [1].

To comparatively assess why previous work has documented contrasting results to the present study, it is useful to look at methodological approaches to study design and training status. For example, a recent study in youth footballers showed 4-min after a pre-load stimulus resulted in the greatest performance when using sport specific tests [15], however, this 4-min recovery condition was compared against recovery durations ranging between just 1–3 min, arguably insufficient to allow physiological recovery following heavy strength repetition lifts [38]. When considering not just potentiation, but also recovery of fatigue prior to match play, longer recovery-durations than 4-min should likely be investigated. When longer recovery durations prior to a 10-m sprint performance test (post-4, post-8, post-12 and post-16 mins) have been assessed in rugby players, it was observed that only 13% of rugby players ran fastest after 4-min of recovery, yet 27% of players achieved their greatest sprint time after 12-min rest and 47% of players ran fastest after 8-min of recovery. Whilst 16-min of recovery was also investigated, only 13% of players produced maximal sprint times in this condition [38]. The observation that most of these rugby players responded best to 8-min (PAP_8_) of recovery could relate to greater (strength) training status of the rugby players tested, when compared to other study population samples investigating PAP. A study conducted by Dello Iacono *et al*. [8] found performance in elite handball players showed the greatest response following 4-min and 8-min recoveries in 10-m and 15-m sprint times. Whether similar findings would be elicited for agility or CMJ performance is unknown. Elite handball players possess higher levels of strength and power across a range of performance measures [41] compared to amateur players, which may be more representative of the current university handball player demographic. It is therefore unknown whether elite professional handball team players would respond best to PAP_8_ rather than PAP_12_ due to greater comparative training status.

Electromyography (EMG) measures were not obtained in the current study. Therefore, we can only speculate on potential muscular and neural mechanisms of action leading to greater test performance. Based on previous work, we would postulate that the pre-load stimulus (3 x 90% 1RM) sufficiently induced greater motor unit excitability, potentially benefiting actions relating to motor unit synchronization or enhanced central input to the motor unit and may have enhanced motor unit excitability [4]. Based on our findings that a 12-min recovery following a standardized pre-load stimulus resulted in greater test performance compared to both PAP_4_ and PAP_8_, it may be that the performance differential was not the difference in potentiation response, but a reduced influence of fatigue, which as previously alluded to is antagonistic in nature with potentiation [39] and likely promoted a better net balance between both potentiation and fatigue responses, likely favourably altering performance [4]. Two secondary outcome variables that were measured in the current study were both Rate of Perceived Exertion (RPE) and heart rate. Both RPE and heart rate are indices of recovery status. Heart rate and RPE were both significantly lower following PAP_12_, *versus* both PAP_4_ & PAP_8_ conditions. The significant reduction in these secondary outcome measures, in combination with greater performance, likely supports the consensus that strength or power performance can only be maximized when potentiation is present alongside enough recovery from fatigue, which in university handball players, was greatest after 12-min of rest.

Previous findings have also found heat to enhance potentiation and alter the effect of acidosis on contractile response of muscle fibers. These observations place some doubt on the role of changes in Ca^2+^ sensitivity in fatigue at physiological temperature. Current testing was performed in temperate environments (18 to 23°C) to accurately simulate match conditions in handball [42]. An active warm-up will internally increase muscle temperature, regardless of ambient heat [27, 43]. The capacity of skeletal muscle to generate speed or power is often maximized when muscle temperature is > 39°C. Therefore, if muscle temperature is not elevated prior to a pre-load stimulus, greater increases in muscle excitability may well occur. It is for this reason that it is important to mirror match warm-up practice when possible to better understand true responses to performance simulations. It is not usually until after 10–20 min of steady state (80–100% of LT) that muscle temperature begins to plateau ~2–4°C above baseline, yet many warm-up protocols employed before PAP interventions likely fail to utilise this important physiological variable when designing warm-ups.

PAP is a highly individualized and complex phenomenon and contrasting results between fatigue and PAP are present within the literature. Tillin and Bishop [7] reported that differences established are mainly related to the pre-load stimulus activity, such as number of sets and repetitions, the recovery period between activities, the intensities, the type of contractions and the loads utilized. Other aspects related to training-status [16, 17], muscle fiber type composition [18], conditioning contraction type [19] and sex [20] have also been reported to affect PAP stimulus. Therefore, to effectively induce a pre-load stimulus it is paramount to establish the optimal relationship between fatigue and PAP, taking the aforementioned variables into account as the relationship between these variables will determine whether or not subsequent performance will be improved, decreased or no different [44]. Current data suggests that females display larger increases compared to males following PAP_12_ (Ishak et al. unpublished data), further highlighting the complexity and individualization of the PAP “phenomenon” and the difficulty in comparing or using previously established data/findings.

## Conclusion

In conclusion, we provide novel data observing that a pre-load stimulus, followed by 12-min of recovery, results in greater maximal jump, sprint and agility measures when compared with a 4-min or 8-min recovery period.

### Practical applications

In line with other recent literature recommendations [15], it is not currently known whether the use of PAP after heavy strength repetitions translates to greater sprint or jump performance during the early part of a handball match. Additionally, depending on position, there is a significant aerobic contribution to handball performance so again it is unclear how the current PAP intervention would translate in assisting an athlete in performing repetitive strength and power movements under fatigue [1]. The efficacy of using PAP as a longer term training stimulus is also a point of interest [38] and is something that would be interesting to assess in future research. It is therefore important for practitioners to identify and establish individualized pre-load stimuli in accordance to each player to ensure an appropriate “recovery window” is provided.

## Supporting information

S1 File (DOCX)
