# Supplementary material for: The influence of recovery period following a pre-load stimulus on physical performance measures in handball players
Source: PLoS One. 2022 Mar 31;17(3):e0249969. doi: 10.1371/journal.pone.0249969 (PMC8970503; doi:10.1371/journal.pone.0249969)
Supplement: S1 File — (DOCX) [file pone.0249969.s001.docx]

| **RPE** | 1 | 2 | 3 | 4 | 5 | 6 | 7 | 8 | 9 | Heart Rate | 1 | 2 | 3 | 4 | 5 | 6 | 7 | 8 | 9 |
| --- | --- | --- | --- | --- | --- | --- | --- | --- | --- | --- | --- | --- | --- | --- | --- | --- | --- | --- | --- |
| **1** | 10 | 13 | 17 | 11 | 13 | 17 | 10 | 12 | 16 | **1** | 132 | 152 | 181 | 129 | 148 | 180 | 128 | 148 | 180 |
| **2** | 12 | 14 | 17 | 12 | 14 | 18 | 11 | 13 | 16 | **2** | 131 | 150 | 181 | 131 | 151 | 180 | 122 | 147 | 177 |
| **3** | 11 | 13 | 18 | 11 | 13 | 18 | 11 | 13 | 17 | **3** | 129 | 147 | 181 | 126 | 147 | 180 | 125 | 149 | 178 |
| **4** | 11 | 13 | 17 | 11 | 14 | 17 | 10 | 12 | 16 | **4** | 131 | 156 | 183 | 129 | 154 | 182 | 125 | 151 | 178 |
| **5** | 12 | 14 | 18 | 11 | 12 | 17 | 11 | 13 | 17 | **5** | 129 | 150 | 184 | 128 | 150 | 183 | 126 | 149 | 180 |
| **6** | 11 | 13 | 18 | 10 | 12 | 17 | 9 | 12 | 17 | **6** | 125 | 153 | 179 | 124 | 155 | 177 | 121 | 144 | 176 |
| **7** | 13 | 15 | 18 | 12 | 15 | 18 | 13 | 15 | 18 | **7** | 125 | 150 | 179 | 125 | 152 | 178 | 120 | 148 | 176 |
| **8** | 11 | 13 | 17 | 10 | 12 | 16 | 9 | 12 | 16 | **8** | 127 | 155 | 181 | 129 | 150 | 182 | 125 | 148 | 179 |
| **9** | 12 | 14 | 18 | 11 | 13 | 17 | 11 | 13 | 17 | **9** | 125 | 151 | 179 | 124 | 152 | 177 | 121 | 152 | 175 |
| **10** | 12 | 14 | 18 | 11 | 13 | 17 | 10 | 12 | 17 | **10** | 128 | 157 | 183 | 127 | 154 | 181 | 125 | 149 | 178 |
| **11** | 11 | 14 | 18 | 11 | 13 | 17 | 11 | 13 | 17 | **11** | 131 | 153 | 183 | 128 | 152 | 181 | 125 | 148 | 179 |
| **12** | 12 | 14 | 18 | 12 | 14 | 17 | 12 | 14 | 17 | **12** | 128 | 147 | 183 | 127 | 153 | 182 | 128 | 145 | 178 |
| **13** | 12 | 14 | 18 | 11 | 14 | 17 | 11 | 13 | 16 | **13** | 128 | 152 | 183 | 128 | 149 | 183 | 126 | 149 | 180 |
| **14** | 11 | 14 | 18 | 11 | 13 | 17 | 10 | 12 | 16 | **14** | 129 | 152 | 184 | 127 | 149 | 181 | 125 | 146 | 178 |
| **15** | 11 | 13 | 17 | 12 | 14 | 18 | 9 | 12 | 16 | **15** | 127 | 152 | 182 | 128 | 147 | 183 | 126 | 143 | 180 |
| **16** | 11 | 13 | 18 | 11 | 13 | 17 | 10 | 13 | 16 | **16** | 127 | 150 | 181 | 128 | 151 | 183 | 128 | 149 | 180 |
| **17** | 12 | 14 | 17 | 10 | 13 | 17 | 10 | 12 | 16 | **17** | 136 | 149 | 184 | 133 | 147 | 183 | 127 | 145 | 178 |
| **18** | 12 | 14 | 17 | 12 | 15 | 18 | 11 | 14 | 17 | **18** | 134 | 152 | 183 | 133 | 150 | 184 | 131 | 148 | 180 |
| **19** | 9 | 13 | 17 | 9 | 13 | 17 | 8 | 12 | 16 | **19** | 127 | 149 | 181 | 126 | 152 | 180 | 125 | 150 | 179 |
| **20** | 11 | 15 | 18 | 11 | 14 | 18 | 10 | 13 | 17 | **20** | 127 | 149 | 180 | 129 | 147 | 181 | 125 | 148 | 179 |
|  |  |  |  |  |  |  |  |  |  |  |  |  |  |  |  |  |  |  |  |
|  |  |  |  |  |  |  |  |  |  |  |  |  |  |  |  |  |  |  |  |
| Jumps | 1 | 2 | 3 |  | Agility | 1 | 2 | 3 |  | Sprint | 1 | 2 | 3 |  |  | Graphs | **1** | **2** | **3** |
| **1** | 49.6 | 50.0 | 50.3 |  | **1** | 8.49 | 8.41 | 8.34 |  | **1** | 3.44 | 3.40 | 3.29 |  |  | Jump | 47.80 | 47.97 | 49.05 |
| **2** | 52.5 | 52.0 | 53.0 |  | **2** | 7.41 | 7.26 | 7.20 |  | **2** | 3.86 | 3.81 | 3.78 |  |  |  | 3.37 | 3.30 | 3.09 |
| **3** | 45.8 | 46.0 | 47.3 |  | **3** | 9.59 | 9.49 | 9.38 |  | **3** | 3.53 | 3.50 | 3.48 |  |  |  |  |  |  |
| **4** | 52.0 | 52.3 | 53.0 |  | **4** | 7.32 | 7.27 | 7.21 |  | **4** | 3.63 | 3.57 | 3.51 |  |  | Agility | 8.33 | 8.28 | 8.19 |
| **5** | 42.3 | 42.0 | 43.0 |  | **5** | 9.31 | 9.23 | 9.13 |  | **5** | 3.31 | 3.34 | 3.20 |  |  |  | 0.87 | 0.88 | 0.87 |
| **6** | 49.7 | 49.0 | 50.0 |  | **6** | 7.42 | 7.27 | 7.15 |  | **6** | 3.68 | 3.65 | 3.61 |  |  |  |  |  |  |
| **7** | 37.7 | 38.0 | 40.0 |  | **7** | 9.55 | 9.41 | 9.35 |  | **7** | 3.54 | 3.45 | 3.32 |  |  | Sprint | 3.52 | 3.51 | 3.40 |
| **8** | 47.5 | 48.1 | 48.8 |  | **8** | 8.92 | 8.87 | 8.71 |  | **8** | 3.58 | 3.57 | 3.35 |  |  |  | 0.21 | 0.20 | 0.21 |
| **9** | 49.5 | 50.0 | 50.5 |  | **9** | 7.42 | 7.28 | 7.15 |  | **9** | 3.18 | 3.22 | 3.11 |  |  |  |  |  |  |
| **10** | 46.7 | 47.0 | 49.0 |  | **10** | 8.61 | 8.68 | 8.57 |  | **10** | 3.65 | 3.63 | 3.51 |  |  |  |  |  |  |
| **11** | 47.0 | 47.3 | 48.3 |  | **11** | 8.86 | 8.88 | 8.72 |  | **11** | 3.39 | 3.43 | 3.37 |  |  |  |  |  |  |
| **12** | 52.1 | 52.0 | 53.0 |  | **12** | 7.53 | 7.39 | 7.35 |  | **12** | 3.82 | 3.75 | 3.68 |  |  |  |  |  |  |
| **13** | 49.0 | 49.1 | 49.4 |  | **13** | 8.22 | 8.24 | 8.09 |  | **13** | 3.61 | 3.67 | 3.45 |  |  |  |  |  |  |
| **14** | 46.0 | 47.0 | 47.8 |  | **14** | 8.78 | 8.80 | 8.75 |  | **14** | 3.23 | 3.20 | 3.09 |  |  |  |  |  |  |
| **15** | 47.0 | 48.0 | 49.2 |  | **15** | 7.10 | 7.10 | 7.08 |  | **15** | 3.35 | 3.33 | 3.30 |  |  |  |  |  |  |
| **16** | 49.6 | 49.4 | 50.0 |  | **16** | 7.40 | 7.38 | 7.21 |  | **16** | 3.32 | 3.35 | 3.10 |  |  |  |  |  |  |
| **17** | 47.5 | 48.0 | 48.7 |  | **17** | 9.41 | 9.34 | 9.22 |  | **17** | 3.68 | 3.59 | 3.35 |  |  |  |  |  |  |
| **18** | 47.3 | 47.0 | 48.6 |  | **18** | 8.33 | 8.33 | 8.23 |  | **18** | 3.25 | 3.29 | 3.15 |  |  |  |  |  |  |
| **19** | 48.7 | 48.1 | 50.0 |  | **19** | 9.39 | 9.39 | 9.27 |  | **19** | 3.56 | 3.56 | 3.50 |  |  |  |  |  |  |
| **20** | 48.5 | 49.0 | 51.0 |  | **20** | 7.55 | 7.58 | 7.42 |  | **20** | 3.87 | 3.95 | 3.76 |  |  |  |  |  |  |
